# Light People: Professor James C. Wyant

**DOI:** 10.1038/s41377-021-00582-x

**Published:** 2021-08-12

**Authors:** Hui Wang, Cun Yu

**Affiliations:** Department of International Cooperation, Changchun Institute of Optics, Fine Mechanics and Physics, Chinese Academy of Sciences, 3888 Dong Nan Hu Road, Changchun, 130033 China

**Keywords:** Optical physics, Optical materials and structures

## Abstract

He is the founding dean of the University of Arizona’s College of Optical Sciences. He was elected president of the OSA and the SPIE, two of the most respected international academic organizations in the world of optics. He is the founder or co-founder of multiple hi-tech companies. He is Prof. James C. Wyant. Professor Wyant is many things: master of optics, entrepreneur, and philanthropist, but above all, he is one of those rare lucky people who see their childhood dreams come true. He is the hardworking scientist who pushes optical science to the limit. He is the successful entrepreneur whose Midas touch turns research achievements into lucrative commercial projects. He is the generous philanthropist who has given a large fortune to help promote the development of optical science. He is the avid athlete who enjoys the outdoors and takes pleasure in hiking and running. From tender memories to cutting-edge research and hard-nosed business advice, Prof. Wyant will share with us how he became fascinated with optics and what he thinks about academic life and organizations.

## Abstract (web summary)

From tender memories to cutting-edge research and hard-nosed business advice, Prof. Wyant shares how he became fascinated with optics and what he thinks about academic life and organizations.

## Graphical abstract

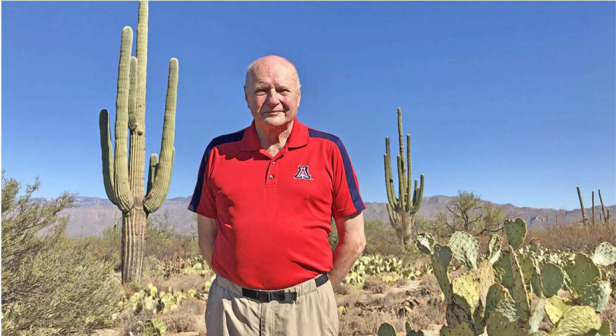



**Biography:** James C. Wyant is a professor emeritus and founding dean at the College of Optical Sciences at the University of Arizona, where he was director (1999–2005), dean (2005–2012), and a faculty member since 1974. He received a B.S. in physics from Case Institute of Technology and M.S. and Ph.D. in optics from the University of Rochester. He was a founder of the WYKO Corporation and served as its president and board chairman from 1984 to 1997 and he was a founder of the 4D Technology Corporation and served as its board chairman from 2002 to 2018. Wyant is a member of the National Academy of Engineering, the National Academy of Inventors, a Fellow of OSA (Optical Society of America) and SPIE (International Society of Optics and Photonics), a distinguished fellow of the Optical Society of India, an honorary member of the Optical Society of Korea, and former editor-in-chief of the OSA journal Applied Optics. He was the 2010 president of OSA and the 1986 president of SPIE. Wyant has received several awards for his technical work, including the OSA Joseph Fraunhofer Award; SPIE Gold Medal; SPIE Technology Achievement Award; SPIE Chandra Vikram Award; SPIE Visionary Award; and five R&D 100 awards. He received the University of Rochester College of Engineering Distinguished Alumnus Award, the Case Alumni Association Gold Medal Award, Case Western Reserve University Athletic Hall of Fame, the University Medal from Case Western Reserve University, and an Honorary Doctor of Science from the University of Rochester. For his entrepreneurial activities, Wyant has received several awards, including Arizona’s “Innovator of the Year” Product Award; the Tom Brown Excellence in Entrepreneurship Award; and the University of Arizona Technology Innovation Award. The University of Arizona renamed the College of Optical Sciences the James C. Wyant College of Optical Sciences in 2019.

**1. In November 2018,** **the University of Arizona received an historic pledge of $20 million** **from you and your family to support 10 new faculty positions in its College of Optical Sciences. This is not the first time you have donated large sums of money to optics research and education, and as far as I know, you’ve been donating in many ways and giving back to the community as much as you can. Could you talk a little bit about what motivates your generosity?**

My primary reason for donating is to show my gratitude for everything my career in optics has given me and to honor my mentors who have helped shaped who I am today. I also hope that my commitment to support optics education will inspire others to do the same.

**2. As a successful entrepreneur with significant expertise in launching and growing industry corporations you have received several awards for your entrepreneurial activities, including Arizona’s “Innovator of the Year” Product Award and the Tom Brown Excellence in Entrepreneurship Award. Could you talk about why you wanted to start the business? What do you think are the biggest challenges for scientists trying to commercialize their research achievements? Any tips or warnings?**

From an early age, I knew I wanted to do three things when I grew up—become a professor, be an inventor, and start a company. As a young professor/researcher, I put together an excellent student research group, and we became very excited about the possibility of turning some of our research into a commercial product.

One of the biggest challenges involved was accessing need and designing a unique product that many people would buy. In addition, we had to have a product that would still operate properly day-after-day-after-day after being shipped halfway around the world and operated by people with many different skill levels.

The best tip I can give anyone trying to commercialize their research is to accept that you cannot do everything well. Acknowledge your strengths and weaknesses, and hire people with skills that you do not have. When I started WYKO, I quickly realized I lacked marketing, writing, and accounting skills, so I hired talented people for those areas.

**Figure:**
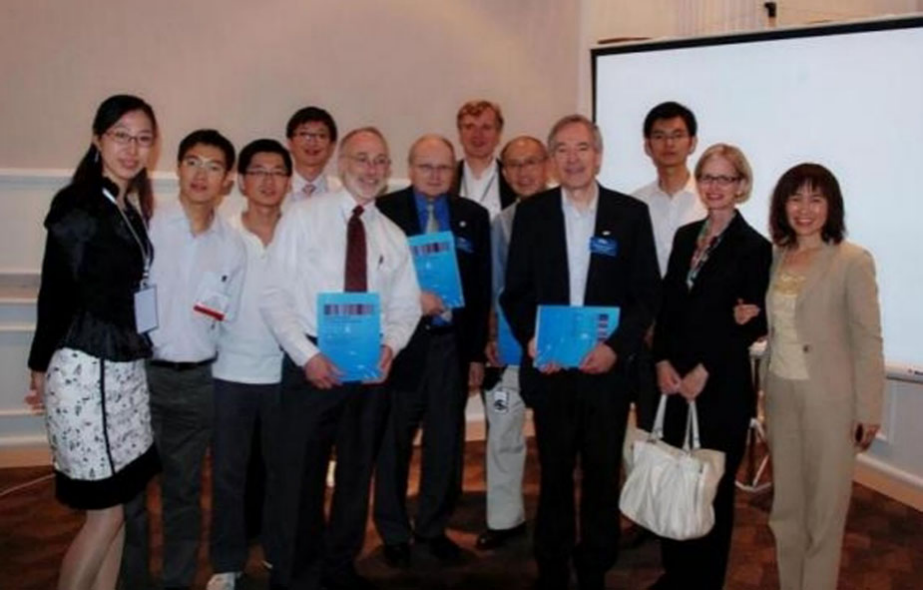
Prof. Wyant with his OSA group met CIOMP delegation at the 8th Pacific Rim Conference on Lasers and Electro-Optics (CLEO) in Shanghai in 2009.

**3. Your entrepreneurial success is firmly rooted in your excellent scientific and technological achievements. Would you care to share with us any moments of joy when you made scientific breakthroughs in your research?**

There were many moments throughout my academic and professional career when a particular concept I was working on just clicked and I suddenly understood the concept in great detail—making a previously unsolved problem clear and obvious. I will give two examples—one technical and one business-related.

One technical example occurred during my time at ITEK in Boston, Massachusetts, in the late 1960s when I was working with computer-generated holograms (CGHs) to test aspheric optics. While I understood mathematics well, I did not totally understand the physics of a CGH. One day I realized that a CGH was just a computer-drawn binary representation of a regular interferogram. After that, I made very rapid progress using CGHs to test aspheric surfaces. In hindsight the solution was obvious, and I often wonder why I did not think of it earlier. I think I spent too much time on the math because I liked math, but it was really important to not just understand the math, but to also think about the physics of what was happening. Without really understanding the physics, I could have never really understood what I was doing. Math by itself, was not enough.

The second example concerns a business decision. When we started the WYKO Corporation the major market we intended to sell metrology equipment to was optical fabrication, and we did sell them a lot of equipment. The magnetic data storage industry also came to us and bought some equipment, but we continued to concentrate our efforts on interferometric metrology equipment for optical manufacturing. One day, while mowing the lawn at my home, I suddenly realized that the market of selling metrology equipment to manufactures of magnetic hard disk drives for the new personal computers was many times greater than the potential market for the optics industry—in particular for measuring the shape of magnetic recording heads used in hard disk drives. After that, we concentrated on designing interferometers and software for the magnetic data storage industry. The hard disk drive industry grew at a fantastic rate, and our business was able to take advantage of this growth. In hindsight, it is obvious that we needed to take advantage of the rapid growth in the hard disk drive industry because they needed metrology equipment very similar to what we were already selling, but at the time we were concentrating on the market that had been good to us in the past, rather than the market that had the best potential future.

**4. Your major research interests involve using interferometric techniques combined with computers and modern electronics to produce “state of the art” solutions for a variety of metrology problems. What were the most significant scientific and technological challenges?**

Back in the early 1980’s when we first started our company, WYKO, the main challenge was the lack of powerful “out-of-the-box” technology that is easily obtained today. Computers were slow with little memory, so we had to write very efficient software to do calculations, Commercial graphic display boards were not available, so we built bit-mapped display boards to accurately show our results. And we had no way to interface solid-state detector arrays to a computer, so we had to build our own electronics to interface between the computers and detectors. I would have loved to have had one of today’s smartphones back then!

**5. It is often said that the stars seen on earth are actually what they looked like millions of years ago. If the distance is 100 light years, then what we see is the light emitted by the constellation 100 years ago. So, are lights seen with high-power telescopes and those seen with naked eyes emitted at the same time? (This question is chosen from a selection of questions from the CIOMP graduate students.)**

Large aperture telescopes collect more light than our small aperture eyes so they can detect stars that are fainter due to being farther away. If the star is 100 light-years away, the light was emitted 100 years ago and has been traveling at the speed of light for 100 years to reach us. Therefore, some of the light seen with high-power telescopes was emitted long before the light seen with naked eyes.

**6. You have served as the president of OSA and SPIE, two world-renowned and influential organizations in the field of optics. What are their similarities and differences please?**

OSA and SPIE are more similar now than they were many years ago. Both organizations are financially sound and managed very well, with excellent leaders and extremely competent staff. While traditionally OSA has been known to be more focused on science and SPIE on engineering, both organizations are now very strong in both science and engineering. Also, both organizations are proud to be international societies, with members, officers, and meetings around the world.

**7. As the 1986 SPIE President and the 2010 OSA President, you have had significant experiences with the leadership, awards, and major meetings of both societies. What responsibilities did these positions involve and what influence did this kind of volunteer work have on your career?**

Before becoming President of SPIE or OSA, I served on numerous committees; was active in both SPIE and OSA journals including being the Editor-In-Chief for OSA’s Applied Optics; and was involved with organizing meetings for both OSA and SPIE. As President of SPIE, I helped to increase our involvement in meetings held outside the United States and worked to expand the size of the exhibits at SPIE meetings. While I was President of OSA, we began publishing a greater number of specialized optics journals and we increased our international activities and membership. I was fortunate to visit many new OSA student chapters around the world.

**Figure:**
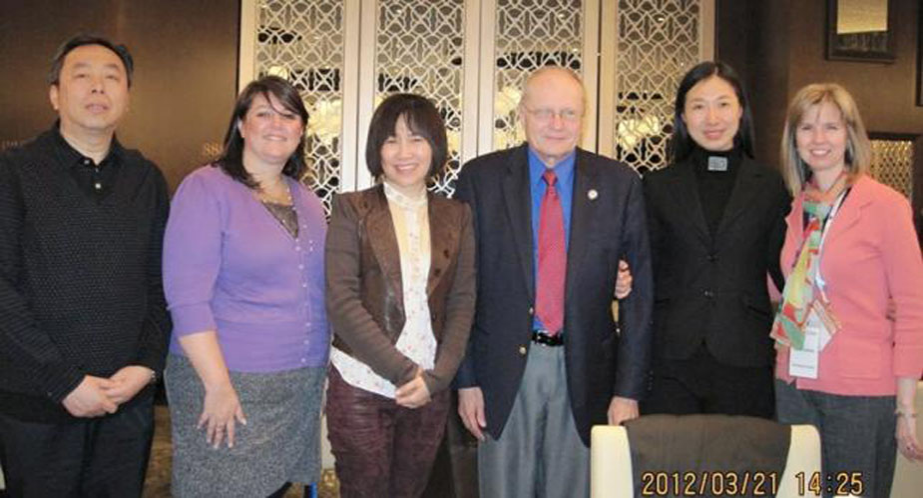
Prof. Wyant with his OSA executives met CIOMP delegation in 2012.

**8. The College of Optical Sciences at the University of Arizona is one of the best optical engineering/science institutes in the world. As the founding dean and a professor emeritus of the College, what qualities do you look for when recruiting new faculty and graduate students?**

It is so important for both faculty and graduate students to be passionate about the field they have chosen, along with being smart and ambitious. Good communication skills are a must, as well as the ability to work well with others. Personally, I have found that graduate students that are also committed to a hobby, such as sports or music, tend to be motivated and have a strong work ethic.

**9. As I know, you have paid three visits to the Changchun Institute of Optics, Fine Mechanics and Physics (CIOMP), Chinese Academy of Sciences, what are your impressions of it?**

I enjoyed my visits to CIOMP and was very impressed with the facilities, faculty, and students. Everyone was very welcoming and went out of their way to show me their labs and introduce me to their research. I found the labs and equipment to be state-of-the-art and the people extremely dedicated.

**Figure:**
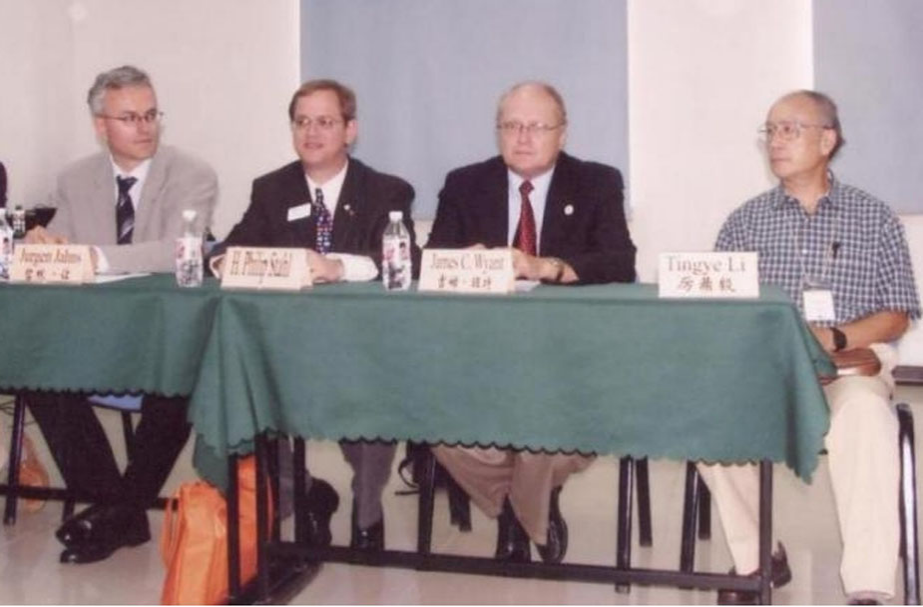
Prof. Wyant participated in the ICO 2005 organized by CIOMP in Changchun.

**Figure:**
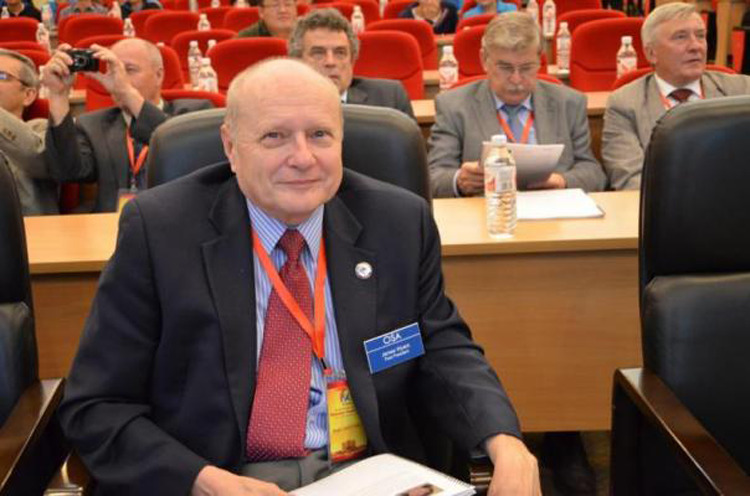
Prof. Wyant participated in the academic conference for celebrating the 60th anniversary of CIOMP.

**Figure:**
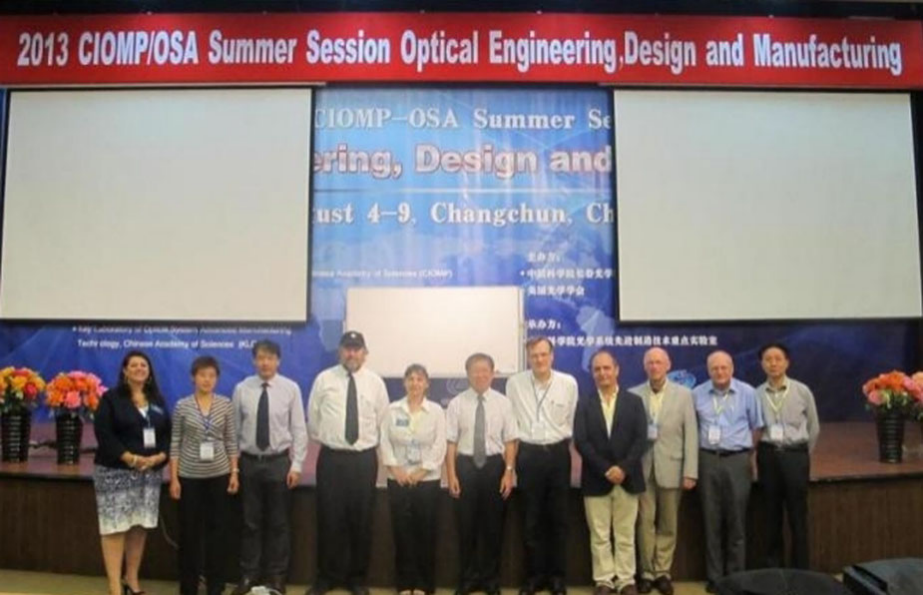
Prof. Wyant participated in the summer school jointly organized by CIOMP and OSA in 2013.

**Figure:**
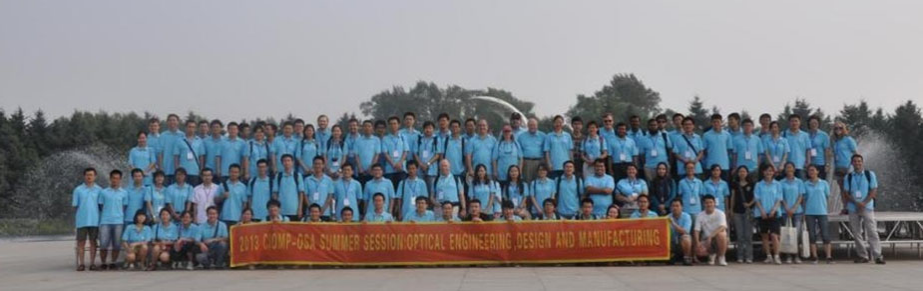
Hiking with with students during summer school in 2013.

**10. You once said, “Optics is a wonderful career where you can be a physicist, an engineer, an entrepreneur, or a combination of the three.” So, when and how did you become interested in optics?**

I first started thinking about optics between my junior and senior years at Case Institute of Technology (CIT). I got a summer job at Libby-Owens-Ford Glass Company in Toledo, Ohio, where I was given an optics project to develop an instrument that would inspect automobile window glass to determine the amount of surface scatter. The project was such a success that the instrument was installed in their factory.

Then during my first senior physics lab at CIT, I saw my first laser and my first hologram. My professor illuminated a cloudy glass plate with a Helium-Neon laser. A stapler appeared that looked so realistic, I actually tried to grab it. I remember thinking that I just had to learn the science behind creating this amazing 3-D image!

**11. From a student to a scientist, a teacher, and an academician, how do you think your attitude toward scientific research has changed over the years?**

When I was a young researcher, I remember there being times when I discarded certain research ideas because they were not practical or useful, but since then I have learned that what might not be practical or useful at the present time may become highly practical when other technology becomes available. And these ideas may be useful for new applications we have not thought of yet.

**Figure:**
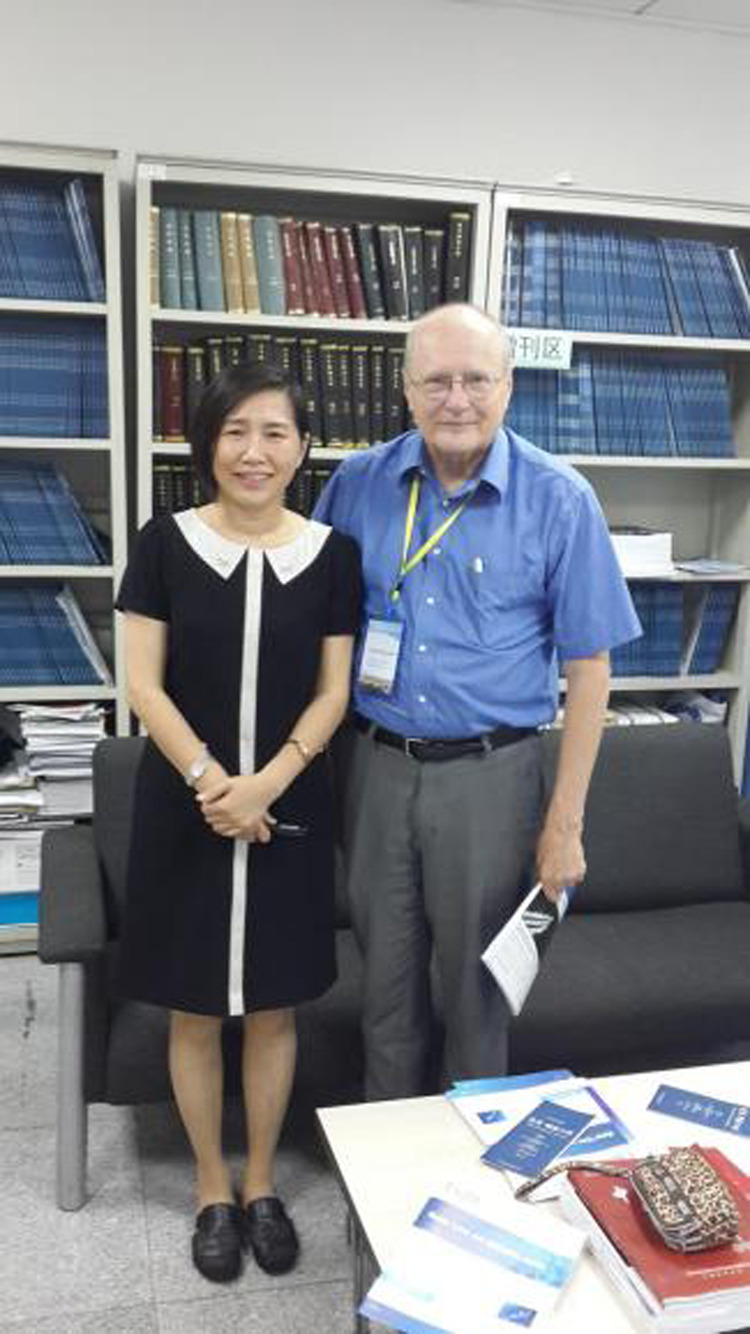
Prof. Wyant and Dr. Yuhong Bai, the director of Light Publishing Group.

**12. I read that you were captain of the cross-country team at Case Institute of Technology (CIT) for which you ran outdoors in winter, even falling down a few times on ice. Is running a hobby of yours? Do you have any other hobbies?**

During my undergraduate years at CIT, I was part of the track and field team, as well as the cross-country team where we ran races outside over natural terrain. Participating in athletics those 4 years was one of the best experiences of my academic career. I learned valuable skills such as how to be part of a team and how to collaborate. The experience also helped me develop a habit of exercise that I still maintain today, although mostly walking and hiking now.

My other big hobby is ham radio. I have been a ham radio operator for more than 60 years which I find interesting because there are many similarities between radio communications and optics. When I first became interested in holography as an undergraduate student, I realized how much my knowledge of radio communications helped me understand the concepts of optics. Then, during this past year, when I had to stay home more due to the pandemic, I knew I was going to need a project. For several years, I had been thinking about putting together an Earth-Moon-Earth (EME, “Moon Bounce”) station to bounce radio waves off the moon to communicate with operators around the world. While designing this station, I realized that my knowledge of optics now helped me understand many aspects of EME.

**Figure:**
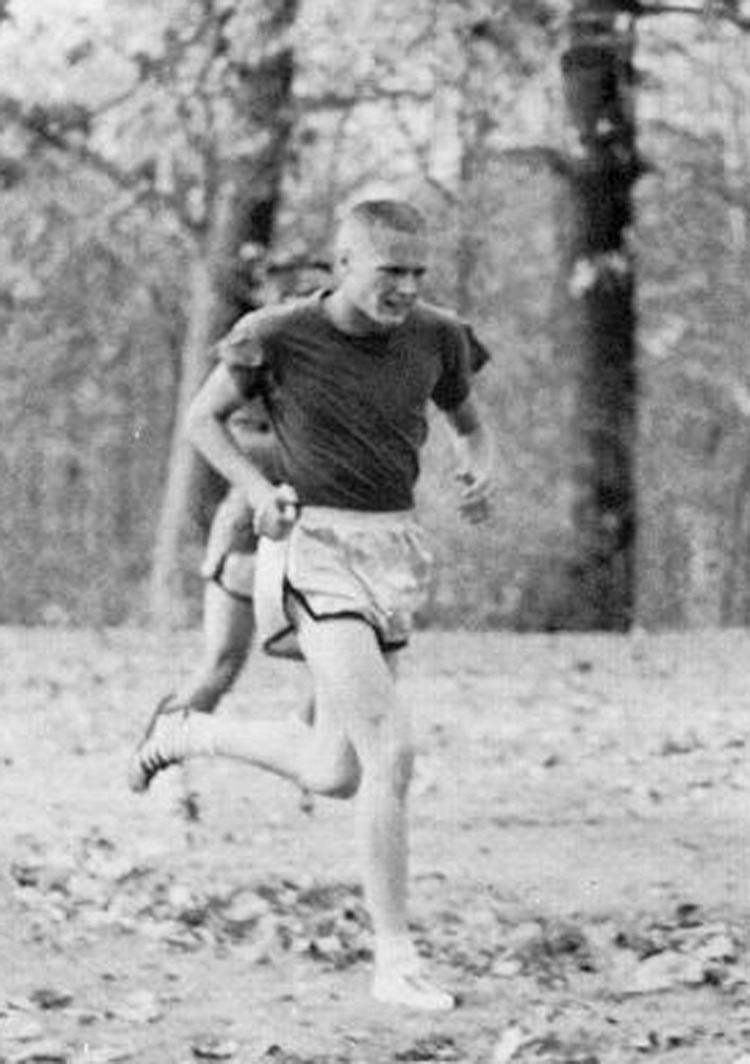
James C. Wyant was running as a junior in college in 1963.

**13. If you could travel in time, which period in history or in your life would you like to go back to? Why?**

My father was killed in a farming accident when I was 5 years old, therefore most of my memories of him are ones told to me by my mother and other family members. I think it would be great to go back to the time before he died (prior to 1948) so I could commit to memory his personality and interactions with me during those first years of my life, especially since I am often told that I am very much like my father. Before his death, he was known for his clever inventions used on our farm.

**14. Could you tell us who you consider as your mentor in your career and how they have influenced you?**

I always say that the one person that influenced my career choices the most was Bob Shannon. I first met him when I was a graduate student at the University of Rochester and, upon graduation, he hired me as an optical engineer at ITEK Corp. in Boston, Massachusetts. Shortly afterward, Bob left ITEK to become a professor at the Optical Sciences Center (OSC) at the University of Arizona. Five years later, Bob encouraged me to leave the industry and join the faculty at OSC. Bob also encouraged my involvement with the professional societies OSA and SPIE.

**Figure:**
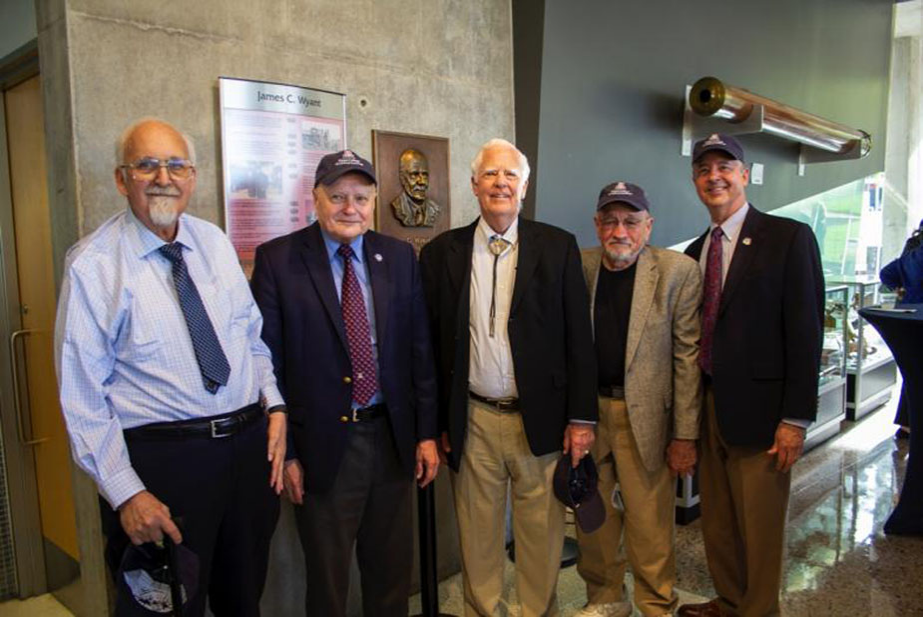
Group photo at the College of Optical Sciences Renaming event (from left to right: Harry Barrett, James C. Wyant, Dick Powell, Bob Shannon, and Tom Koch).

**15. How do you balance your work and family life?**

To be honest, when I was young and in the prime years of my career, I probably did not balance work and family life as well as I could have. For years, I worked 7 days a week, almost never taking a vacation. Now that I am retired, I realize all that hard work paid off and I can now “play” 7 days a week, but I still in some ways wish I had always had a better life/work balance.

**16. What advice do you have for young researchers?**

Find your passion and, once you do, surround yourself with smart people who will teach and inspire you to push yourself. A lot of the time, a student that has just received their degree will accept a job with the company that offers them the most money. While money certainly should be a consideration, it is far more important at that point in their career to accept the job that offers challenging projects you would be excited to be working on.

I also think it is very important to join and be involved with professional societies. Contacts you make by attending meetings and being on committees may prove to be invaluable for networking. In addition, some of those you meet may become professional mentors or lifelong friends.

**Light special correspondent**

*Hui Wang is the Deputy Director of the Office of International Cooperation in the Changchun Institute of Optics, Fine Mechanics and Physics (CIOMP), Chinese Academy of Sciences (CAS). She currently works on international communication and cooperation for the CIOMP and was a founding member for the Nature Publishing Group and CIOMP joint journal Light: Science & Applications. She is the founder of “Rose in Science” and has published several articles in Acta Editologica, International Talent, Light: Science & Applications, etc., and was invited to take an interview by SPIE Women in Optics, which was published in 2015.* 

*Cun Yu works at the Office of International Cooperation in the Changchun Institute of Optics, Fine Mechanics and Physics (CIOMP), Chinese Academy of Sciences (CAS). Her main duties cover the Sino-Belarus International Innovation Center, the CIOMP English website, international cooperation projects, and exchanges between CIOMP and institutions in Belarus, Ukraine, and Russia. She has published multiple articles in the journal International Talent, and is a member of CSA’s Science & Technology Translations Association*.

